# A Novel Hybrid Deep Neural Network to Predict Pre-impact Fall for Older People Based on Wearable Inertial Sensors

**DOI:** 10.3389/fbioe.2020.00063

**Published:** 2020-02-12

**Authors:** Xiaoqun Yu, Hai Qiu, Shuping Xiong

**Affiliations:** ^1^Department of Industrial and Systems Engineering, Korea Advanced Institute of Science and Technology, Daejeon, South Korea; ^2^CETHIK Group Corporation Research Institute, Hangzhou, China

**Keywords:** fall risk, pre-impact fall, deep neural network, machine learning, inertial sensor

## Abstract

Falls in the elderly is a major public health concern due to its high prevalence, serious consequences and heavy burden on the society. Many falls in older people happen within a very short time, which makes it difficult to predict a fall before it occurs and then to provide protection for the person who is falling. The primary objective of this study was to develop deep neural networks for predicting a fall during its initiation and descending but before the body impacts to the ground so that a safety mechanism can be enabled to prevent fall-related injuries. We divided the falling process into three stages (non-fall, pre-impact fall and fall) and developed deep neutral networks to perform three-class classification. Three deep learning models, convolutional neural network (CNN), long short term memory (LSTM), and a novel hybrid model integrating both convolution and long short term memory (ConvLSTM) were proposed and evaluated on a large public dataset of various falls and activities of daily living (ADL) acquired with wearable inertial sensors (accelerometer and gyroscope). Fivefold cross validation results showed that the hybrid ConvLSTM model had mean sensitivities of 93.15, 93.78, and 96.00% for non-fall, pre-impact fall and fall, respectively, which were higher than both LSTM (except the fall class) and CNN models. ConvLSTM model also showed higher specificities for all three classes (96.59, 94.49, and 98.69%) than LSTM and CNN models. In addition, latency test on a microcontroller unit showed that ConvLSTM model had a short latency of 1.06 ms, which was much lower than LSTM model (3.15 ms) and comparable with CNN model (0.77 ms). High prediction accuracy (especially for pre-impact fall) and low latency on the microboard indicated that the proposed hybrid ConvLSTM model outperformed both LSTM and CNN models. These findings suggest that our proposed novel hybrid ConvLSTM model has great potential to be embedded into wearable inertial sensor-based systems to predict pre-impact fall in real-time so that protective devices could be triggered in time to prevent fall-related injuries for older people.

## Introduction

Falls are a major safety concern for the older people. Annual fall rates range from 30% among those aged over 65 years old to 50% for those over 85 (Rubenstein, 2006). Due to the high prevalence, falls are the leading cause of both fatal and non-fatal injuries among the older people (Bergen, 2016). The annual medical costs for falls of the older adults have been estimated at $31.3 billion in United States since 2015 (Burns et al., 2016). Fall-related injuries are considered as “Global Burden of Disease” by the World Health Organization (Murray et al., 2001). Aside from the physical injury, falls can also cause post-fall syndrome such as fear of falling and depression among the elderly (Fleming and Brayne, 2008; Qiu and Xiong, 2015). Therefore, effective fall prevention is critical to mitigate the negative consequences of falls for the older people.

Much work has been done on developing context-aware systems and wearable devices for post-fall detection so that timely medical assistance can be initiated for the older fallers to avoid losses caused by “long-lie” (Özdemir and Barshan, 2014; Yang et al., 2016). However, this approach is reactive since injuries from impact falls have happened already. Recently, researchers have shifted their efforts to a proactive approach-fall prevention, which is performed through fall risk assessment and intervention where the older individuals with high fall risks can be screened out earlier and then treated with appropriate interventions to reduce the risks of future falls (Choi et al., 2017; Qiu et al., 2018). However, the developed fall risk assessment tools and fall intervention programs are mainly focused on predicting and reducing the overall risk of falling in a long period (typically 1 year or more), not for the sudden falls. Many falls in the elderly happen suddenly and are difficult to prevent due to the complex multifactorial nature of falls and inevitably increased fall risks with the elderly as their physical and cognitive abilities deteriorate.

Pre-impact fall prediction can overcome aforementioned limitations of post-fall detection and overall long-term fall risk assessment and intervention. Pre-impact fall refers to a stage after the fall initiation but before the body-ground impact (Hu and Qu, 2016). Therefore, this method can predict sudden falls before the body hits against the ground (e.g., pre-impact), which make it possible to timely activate on-demand fall protection systems such as wearable airbags to prevent fall-related injuries. Because of very short period of falling (around 800 ms) and various types of falls (Sucerquia et al., 2017; Tao and Yun, 2017), to predict the fall before the ground impact accurately under different scenarios is very challenging and worthy of research investigation. Some researchers have recently attempted to tackle this challenge using different approaches (Lee et al., 2015; Sabatini et al., 2016; Li M. et al., 2018; Zhong et al., 2018; Ahn et al., 2019). In general, wearable sensors or environmental cameras were utilized and simple threshold-based algorithms were developed to predict pre-impact falls using some selected fall indicators related to human motions. Even though threshold-based algorithms are easy to implement due to simple structure and low computation cost, the thresholds are highly dependent on the certain types of falls (e.g., forward fall, backward fall) and the tested subjects, which can not fit well for other fall types (lateral fall, vertical fall, etc.) and different older individuals in the real-world. In other words, threshold-based algorithms lack the generalizability and thus are difficult for practical applications. A few studies utilized conventional machine learning methods such as Support Vector Machine and Fisher Discriminant Analysis to predict pre-impact falls (Aziz et al., 2014; Liang et al., 2018; Wu et al., 2019). Tested by small amount of data from very limited types of simulated falls (≤7), they reported good prediction accuracy and reasonable lead time. However, conventional machine learning methods heavily rely on hand-crafted features, which are usually shallow and restricted by human domain knowledge (Wang et al., 2019). Therefore, these approaches generated undermined prediction performance on complex and various falls in the real world as researchers have reported at least 15 common fall types and 19 activities of daily living (ADL; Sucerquia et al., 2017; Tao and Yun, 2017).

Very recently, with the fast advancement of deep learning and computing hardware, a few studies explored deep neural network based algorithms for pre-impact fall prediction. Li et al. (2019) applied convolutional neural network (CNN) on RGB image data recorded by Kinect for pre-impact fall prediction during gait rehabilitation training. Even though they achieved a prediction accuracy of 100% within 0.5 s after a fall initiation, they only tested the model on one type of fall and normal walking. Tao and Yun (2017) proposed a long short term memory (LSTM) model using skeleton data captured by Kinect to predict pre-impact fall. The developed model showed high sensitivity (91.7%) but relatively low specificity (75%), indicating that the model could recognize most of pre-impact falls but with high false alarm rate. Both high sensitivity and specificity are essential for the practical applications. In addition, this method is only restricted in home environment due to the limitations of stationary settings that Kinect cameras often suffer from. Torti et al. (2018) applied an overlapping sliding window segmentation technique to label falling process into three stages (non-fall, pre-impact fall or alert, and fall) and utilized a LSTM model to perform three-class classification based on the SisFall dataset (Sucerquia et al., 2017). They achieved high classification accuracy on fall (98.7%) but lower accuracy on non-fall (88.4%) and pre-impact fall (91.1%), which showed that their algorithm missed ∼9% pre-impact falls and misclassified many non-fall activities as other two classes (most of instances are labeled as non-fall activities in the SisFall dataset due to rarity of fall incidents). Furthermore, both studies only applied one deep learning model-LSTM, comparisons with other deep learning structures were not conducted.

This study aims to develop deep learning algorithms for predicting pre-impact fall in real-time so that a safety mechanism can be enabled to prevent fall induced injuries. A novel hybrid deep neural network which integrates CNN and LSTM architectures was proposed and evaluated on SisFall, a large public dataset of various falls and ADL acquired with accelerometer and gyroscope sensors. We also compared our proposed hybrid model with CNN and LSTM models in terms of model accuracy, latency and learning curve, which could provide more insights about the characteristics of different deep learning models in predicting pre-impact falls. The developed hybrid model is expected to be embedded into wearable inertial sensor based systems, which would be promising to predict pre-impact fall in real-time so that the protective device could be triggered in time to prevent fall-induced injuries for older people.

## Materials and Methods

### Dataset and Labeling

SisFall, a fall and movement dataset with various falls and ADLs acquired with wearable inertial sensors of accelerometer and gyroscope at a frequency of 200 Hz (Sucerquia et al., 2017), was selected for developing and evaluating deep learning algorithms due to two major reasons. First, it is a publicly available dataset which consists of 15 fall types, 19 ADLs and 38 subjects, including the largest amount of data in terms of number of subjects and number of activities (Musci et al., 2018) when compared with other public datasets such as MobiFall (Vavoulas et al., 2014) and UMAFall (Casilari et al., 2017). Second, the protocol is validated by a medical staff and there are 15 older subjects out of total 38 subjects in the SisFall dataset. Thus, the data pattern in SisFall dataset should be close to the real-life ADLs and fall scenarios of the older people.

To be consistent with the earlier studies, we adopted the same criteria as Musci et al. (2018) for labeling data associated with three classes of events.

1. Non-fall: the time interval when the person is performing ADLs.
2. Pre-impact fall or alert: the time interval in which the person is transiting from a controlled state to a dangerous state which may lead to a fall.
3. Fall: the time interval when the person is experiencing a state transition that leads to a fall.

**FIGURE 1:**
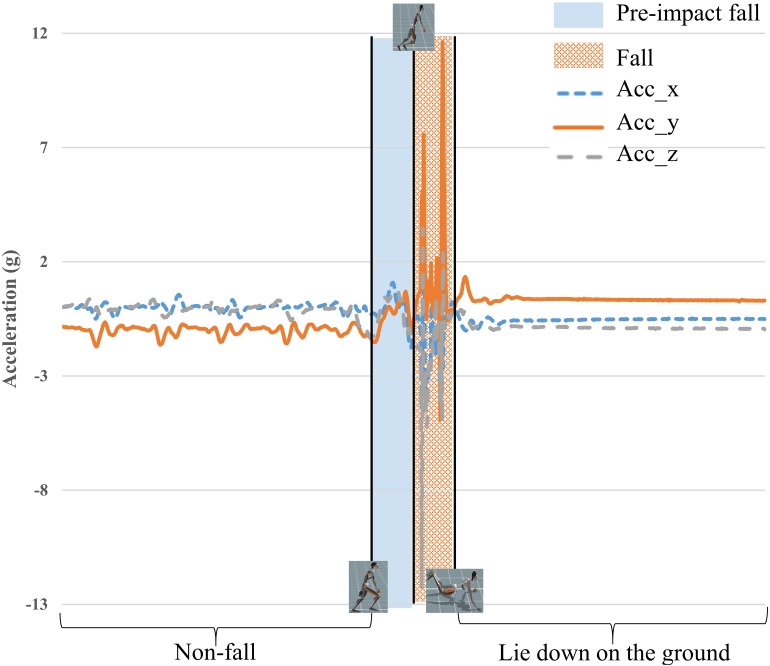
Illustration of labeling three classes during a fall. The beginning period is labeled as non-fall and the blue and orange areas indicate pre-impact fall and fall, respectively; the remainder of the sequence is removed for the labeling.

One representative diagram for three classes of events is illustrated in Figure 1, which shows the 3-axis acceleration data of a forward fall while walking due to a slip. The last part of data is removed for labeling because it is the state after the fall incident.

### Design of Model Architecture

In this study, three models were applied to perform the classification. These models are a CNN model, a LSTM model and our proposed hybrid ConvLSTM model. As shown in Table 1, the CNN model consists of three convolutional blocks and two fully connection layers. Each convolutional block includes convolutional operation, batch normalization, relu and max pooling. The LSTM model follows the similar design as Musci et al. (2018), which consists of LSTM cells, relu, dropout and fully connected layers.

**TABLE 1:** The design of CNN model.

**Type**	**Operations**	**Filter shape**	**Input size**
Conv1	conv	3 × 64	256 × 6
	batchNorm		
	relu		
	max pooling	3 × 64	
Conv2	conv	3 × 64	127 × 64
	batchNorm		
	relu		
	max pooling	3 × 64	
Conv3	conv	3 × 64	62 × 64
	batchNorm		
	relu		
	max pooling	3 × 64	
FC1	fully connection	1920 × 512	1 × 1920
FC2	fully connection	512 × 3	1 × 512
Softmax	softmax	Classifier	1 × 3

The architecture of our proposed ConvLSTM model mainly combines convolutional and recurrent layers. The specific structure of ConvLSTM was determined by the hyperparameter tuning. For this task, we mainly considered three levels of the width (output channels in each convolutional and LSTM layer), two different numbers of layers for both convolutional and LSTM structures, and two levels of dropout (probability of a neuron to be ignored during training). Table 2 summarizes the results of hyperparameter tuning experiments on one training-testing split.

**TABLE 2:** Results of hyperparameter tuning for the structure of ConvLSTM model.

**No.**	**Width**	**No. of Conv layers**	**No. of LSTM layers**	**Dropout**	**Sensitivity (%)**		
					**Non-fall**	**Pre-impact Fall**	**Fall**
1	32	2	2	0.5	88.99	93.31	96.31
2	32	2	2	0.8	91.49	93.31	96.31
3	32	2	4	0.5	91.64	91.21	96.77
4	32	2	4	0.8	92.84	90.79	96.31
5	32	4	2	0.5	92.41	89.12	96.77
6	32	4	2	0.8	90.51	93.72	96.77
7	32	4	4	0.5	94.84	89.54	94.47
8	32	4	4	0.8	91.28	90.38	95.85
9	64	2	2	0.5	90.93	91.63	97.70
10	64	2	2	0.8	91.65	92.89	97.24
11	64	2	4	0.5	88.54	92.05	98.16
12	64	2	4	0.8	85.78	93.51	97.24
13^∗^	64	4	2	0.5	92.30	93.30	95.86
14	64	4	2	0.8	90.18	91.63	96.77
15	64	4	4	0.5	91.47	89.94	95.85
16	64	4	4	0.8	90.22	89.54	96.31
17	128	2	2	0.5	91.73	93.31	93.55
18	128	2	2	0.8	93.77	88.28	95.85
19	128	2	4	0.5	90.58	92.89	96.31
20	128	2	4	0.8	92.10	90.79	98.16
21	128	4	2	0.5	90.45	94.98	96.31
22	128	4	2	0.8	90.75	91.63	99.08
23	128	4	4	0.5	88.85	94.56	95.85
24	128	4	4	0.8	88.53	89.96	97.24

*^∗^Finalized structure of ConvLSTM model.*

**FIGURE 2:**
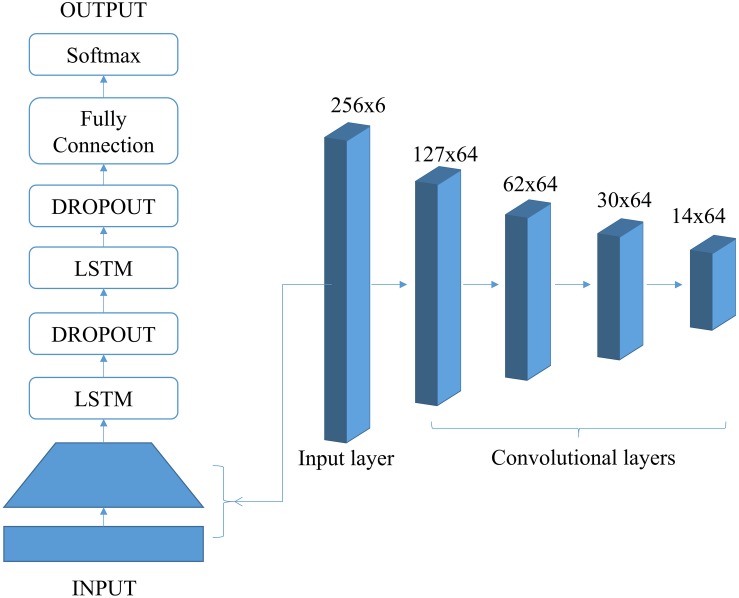
The architecture design of hybrid ConvLSTM model.

As shown in Figure 2, the finalized ConvLSTM structure after hyperparameter tuning consists of four convolutional blocks and two LSTM cells with dropout operations. Each convolutional block contains operations of convolution, batch normalization, relu, and max pooling. The convolutional layers act as feature extractors and provide abstract representations of the input sensor data in feature maps. They could capture short-term dependencies (spatial relationship) of the data. The recurrent layers deal with the long-term temporal dynamics of the activation of the feature maps and identify useful features over time domain in sequential data. More importantly, this structure could integrate advantages of CNN and LSTM on accuracy and efficiency. In the CNN, features are extracted and then used as inputs of fully connected network for classification. However, it ignores long-term temporal relationships in the time sequence, which is important for identifying actions or behaviors. On the contrary, the LSTM uses the memory cell to learn long-term temporal dependencies for the time-series data. However, it is time consuming for running LSTM model due to its complex structure. In the ConvLSTM, CNN layers extract features from the raw data and send to LSTM layers for identifying temporal relationships, which could save time for computing when compared with LSTM model. It is expected that ConvLSTM will outperform both CNN and LSTM models for predicting different fall stages since it can capture both short-term and long-term dependencies of the motion data.

### Model Training

The architectures described in section “Design of Model Architecture” were implemented using the PyTorch library on a computer running Window 10 (64-bit). The models were trained and tested on this computer, equipped with a 3.6 GHz CPU i7-7700, 16GB RAM, and an Nvidia GTX 1080Ti GPU card. Considering the practical applications in the future, we also implemented the models on a microcontroller unit, Jetson Nano (Nvidia, 2019) which runs in Ubuntu 18.04 and equipped with a 64-bit Quad-core ARM A57 at 1.43 GHz CPU, 4GB RAM, and 128-core NVIDIA Maxwell at 921 MHz GPU. During the training, the input data has six dimensions including three-axis accelerometer and three-axis gyroscope. The batch size is 64 and the total epoch is 200. The learning rate is set as 0.0005 and the loss function uses focal loss (Lin et al., 2017).

In order to assess the generalizability of proposed models and prevent overfitting on one specific train/test split, fivefold cross validation was used. There are 23 young and 15 older subjects in SisFall dataset. In our experiment, older subjects were randomly divided into five groups and each group included three older subjects. Young subjects were also randomly divided into five groups in where three groups had five subjects and remaining two groups have four subjects. Each group of older subjects would be randomly combined with one group of young subjects as onefold. Therefore, there were total fivefold for the dataset. Each fold would be the test set and the rest fourfold would be considered as the training set. The ratio between the training and test set was around 80%/20%. By this splitting, we could prevent the same subject appearing in both the training and test sets and maintain the homogeneity among folds at the same time.

All experiments were implemented for 200 epochs and all general hyper-parameters were set exactly same among three deep learning models for a fair comparison. In order to balance classification accuracy of three classes but without losing our focus on the pre-impact fall, the results of the epochs whose summation sensitivity for three classes are within top three and summation sensitivity is the highest for non-fall and pre-impact fall were used for averaging the fivefold cross-validation results. Because the accuracy can be biased by the majority class when the dataset is highly imbalanced, sensitivity instead of accuracy was used as the criteria to determine the best model (Bekkar et al., 2013).

Torti et al. (2018) sets baseline for our study because they also performed three-class classification (non-fall, pre-impact fall, fall) based on the SisFall dataset.

## Results

### Classification Performance

The classification performance is represented by different metrics including sensitivity, specificity and accuracy, which are calculated by equations 1, 2, and 3, respectively.

(1) $$\mathrm{Sensitivity}=\frac{\text{TP}}{\mathrm{TP}+\mathrm{FN}}$$

(2) $$\mathrm{Specificity}=\frac{\text{TN}}{\mathrm{TN}+\mathrm{FP}}$$

(3) $$\mathrm{Accuracy}=\frac{\mathrm{TP}+\mathrm{TN}}{\mathrm{TP}+\mathrm{FP}+\mathrm{TN}+\mathrm{FN}}$$

where TP (True Positives) of non-fall is all non-fall instances that are correctly classified as non-fall class; FN (False Negatives) of non-fall is all non-fall instances that are not correctly classified as non-fall class; TN (True Negatives) of non-fall is all instances of other two classes are not classified as non-fall class; FP (False Positives) of non-fall is all instances of other two classes are wrongly classified as non-fall class. To find the four terms for other two classes, we could replace non-fall with pre-impact fall or fall.

Table 3 summarizes the classification performances of three deep learning models along with the baseline study. The results showed that CNN model had the poorest performance with the mean accuracies of 90.01, 91.51, and 98.38% for non-fall, pre-impact fall and fall, respectively. LSTM model demonstrated higher accuracies (91.59, 93.98, and 97.52%) than CNN, and our proposed hybrid ConvLSTM model achieved the highest accuracies on all classes (93.22, 94.48, and 98.66%). With respect to the sensitivity, the results showed that ConvLSTM model had the mean sensitivities of 93.15, 93.78, and 96.00% for non-fall, pre-impact fall and fall, respectively, which were higher than CNN (89.90, 90.33, and 93.76%) and LSTM models (91.50, 91.48, and 96.22%) except the fall class. For the specificity, the ConvLSTM model had the mean specificities of 96.59, 94.49, and 98.69% for non-fall, pre-impact fall and fall, respectively, which were higher than both LSTM (95.93, 94.00, and 97.54%) and CNN models (95.05, 91.52, and 98.42%).

**TABLE 3:** Classification results of three deep learning models on the test dataset.

	**Class**	**CNN**	**LSTM**	**ConvLSTM**	**Torti et al., 2018**
Sensitivity (%)	Non-fall	89.90	91.50	93.15	88.39
	Pre-impact fall	90.33	91.48	93.78	91.08
	Fall	93.76	96.22	96.00	98.73
Specificity (%)	Non-fall	95.05	95.93	96.59	97.85
	Pre-impact fall	91.52	94.00	94.49	90.77
	Fall	98.42	97.54	98.69	97.93
Accuracy (%)	Non-fall	90.01	91.59	93.22	93.12
	Pre-impact fall	91.51	93.98	94.48	90.93
	Fall	98.38	97.52	98.66	98.33

### Learning Curve

**FIGURE 3:**
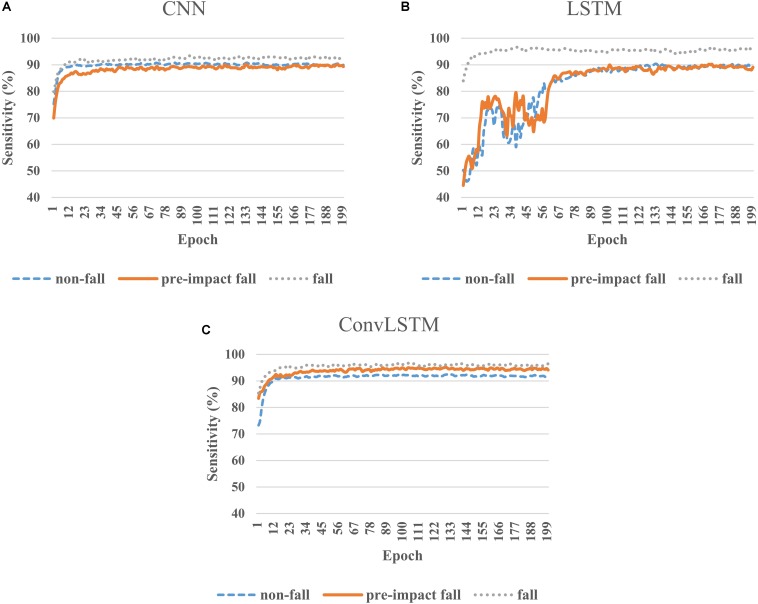
Learning curves of CNN **(A)**, LSTM **(B)**, and ConvLSTM **(C)** models on the training dataset.

Figure 3 presents the representative learning curves of three deep learning models on the same training set. All three models converged after certain number of epochs. Both CNN and ConvLSTM models can quickly learn and achieve the stable status (Figures 3A,C) while LSTM model needs more time to train (Figure 3B). In terms of training sensitivity, the performance of CNN was similar to LSTM on non-fall and pre-impact fall classes. The sensitivities of both models on these two classes fluctuated around 90%; while for the fall class, LSTM model was obviously better than CNN model (Figures 3A,B). For ConvLSTM model, the learning curves on all three classes were above 90%, especially for the pre-impact fall class (Figure 3C).

**FIGURE 4:**
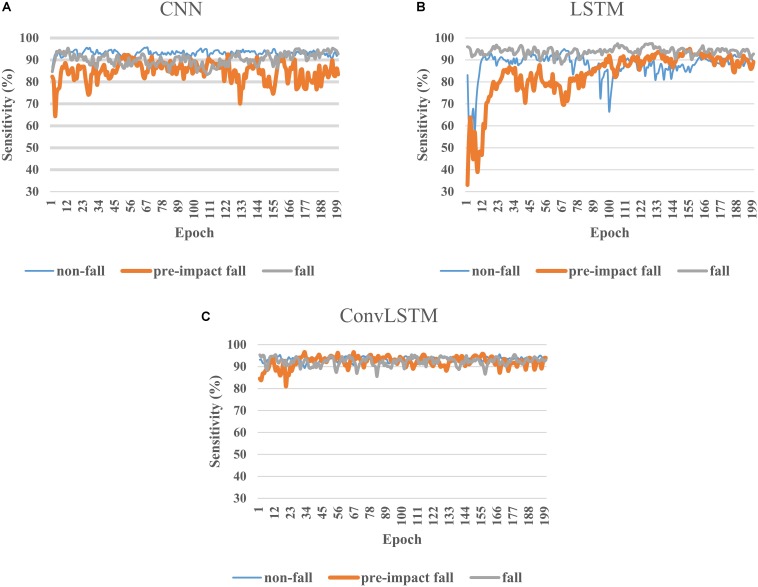
Learning curves of CNN **(A)**, LSTM **(B)**, and ConvLSTM **(C)** models on the test dataset.

Figure 4 depicts the representative learning curves of three deep learning models on the same test set. CNN model failed to learn the features of pre-impact fall data well because there was a large fluctuation on sensitivity even at the end of training (Figure 4A). Figure 4B shows that LSTM model can gradually learn the features of three classes and achieved good sensitivity in the last 50 epochs. Compared to the LSTM model, ConvLSTM model can perform well after only first 20 epochs and maintain the high sensitivity for all three classes until the end of training (Figure 4C).

### Model Latency

The latencies were evaluated with the same training and test sets among three deep learning models. For the practical applications, only processing time on each instance in the test set was summed and averaged over 200 epochs. Models tested on the computer showed average latencies of 0.61, 0.70, and 0.97 ms for CNN, ConvLSTM, and LSTM models, respectively. Further tests on a microcontroller unit (Nvidia Jetson Nano) showed the averaged latency of ConvLSTM model was 1.06 ms, which was slightly higher or comparable with CNN model (0.77 ms) but much lower than LSTM model (3.15 ms).

## Discussion

We developed a hybrid deep learning model (ConvLSTM) that integrates the CNN and LSTM architectures to predict the pre-impact fall from accelerometer and gyroscope sensor data. The performance of this hybrid model was comprehensively compared with CNN and LSTM deep learning models. The experimental results showed that the hybrid ConvLSTM model outperformed CNN and LSTM models in terms of sensitivity, specificity and overall accuracy. The hybrid ConvLSTM model obtained ∼2% higher sensitivities than LSTM and ∼3% higher sensitivities than CNN for all three classes except the fall class. Considering our study aimed to predict the pre-impact fall accurately for preventing fall induced injuries, the high sensitivities for non-fall and pre-impact fall were of significant importance in two perspectives. On the one hand, higher classification sensitivity on non-fall class reflected lower false alarm rate and 2% improvement was very meaningful because dominant instances in the SisFall dataset and real-world scenarios are non-falls or ADLs, and fall instances are very rare. On the other hand, higher classification sensitivity for the pre-impact fall directly indicated the superiority of the ConvLSTM model. In addition, the ConvLSTM model obtained the highest specificities for non-fall (96.59%), pre-impact fall (94.49%), and fall (98.69%) among three deep learning models. A more detailed investigation showed that although the difference on the specificity between ConvLSTM model and LSTM model was marginal, both models had ∼3 and 2.5% higher specificities on pre-impact fall prediction than CNN model. This result indicated that CNN model had the highest rate of misclassifying other two classes as pre-impact fall.

It is understandable that the hybrid ConvLSTM model outperformed individual CNN or LSTM models. CNN could capture local dependency of human motion data (Zeng et al., 2014). For the given time point, the neighboring accelerometer and gyroscope readings are likely to be correlated. However, this dependency is short-term due to the constraint by the size of convolutional kernels (Li F. et al., 2018). On the contrary, LSTM with memory cells could learn to store and output information based on the training, easing the learning of long-term time dependency of motion data (Hochreiter and Schmidhuber, 1997). Therefore, integration of both short-term and long-term dependencies could enhance the ability to distinguish different fall stages that vary in time span and signal distribution.

Our experimental results indicated that the motion features in the long term were more significant in classifying three fall stages (non-fall, pre-impact fall, fall) than those in the short term. This finding was consistent with those of earlier studies using deep learning approaches for human motion recognition (Yao et al., 2017; Li F. et al., 2018). Long-term motion features were also widely used in the conventional machine learning methods for human movement analysis. For example, Su et al. (2016) achieved high accuracy to distinguish falls from non-falls by extracting twelve time-domain features from angular velocity and angle data into a hierarchical classifier. Similarly, Panahandeh et al. (2013) suggested that long-term features of sensor data such as magnitude-squared discrete Fourier transform coefficient and variance were critical for pedestrian activity classification and gait analysis. Furthermore, researchers reported the classification with an integration of time domain (mean, variance, kurtosis, etc.) and gait temporal features (stride time, stance time, double-limb support, etc.) showed better results to differentiate stroke and other neurological disorders than using them separately (Hsu et al., 2018). Compared with conventional machine learning methods, our proposed deep neural networks can eliminate the need of manually designed motion features and can fully utilize the useful information in the raw data for classification.

Two earlier studies utilized CNN and LSTM alone to predict pre-impact falls (Tao and Yun, 2017; Li et al., 2019). Both studies divided the motion data into non-fall and pre-impact fall, and pre-impact fall included several frames before and after the fall initiation so that they could predict the pre-impact fall. However, the data of remaining part of falling (fall class in the three classifications) was not considered, thus these kinds of simple binary classification models can not be used for predicting the fall class. In addition, both studies only tested classification models on a small dataset with limited types of falls (≤4) and ADLs (≤4). To the best of our knowledge, there was only one published study utilizing the LSTM-based three-class classification model to predict the pre-impact fall based on a large dataset-SisFall (Torti et al., 2018). To compare our proposed deep learning models with this baseline study, we also used the SisFall dataset and strictly followed the same criteria for labeling three different classes. Comparison to the benchmark (Table 3) showed that our hybrid ConvLSTM model achieved higher sensitivity of ∼5 and 3% for the non-fall and pre-impact fall, respectively, indicating considerably lower false alarm rate but higher true alarm rate for the pre-impact fall prediction. However, our ConvLSTM model had ∼3% lower sensitivity than the benchmark on predicting the fall class. This could be caused by the different strategy we used to choose the best model. We prioritized the high sensitivity on classes of non-fall and pre-impact fall because the primary objective of this study was to predict a fall with a reasonable lead time before the body impacts to the ground rather than detect a fall after it happens. For the specificity, even though there was no considerable difference on classes of non-fall and fall, our ConvLSTM model outperformed the benchmark on the class of pre-impact fall (higher specificity by 3.7%), which demonstrated lower misclassification rate on pre-impact fall prediction.

In terms of latency, LSTM model is time consuming due to its complex structure and difficulty in parallel computing. In the proposed hybrid ConvLSTM model, the first CNN layers which are appropriate for parallel computation would extract features hierarchically from the raw motion sensor data. The extracted features would be inputted to following LSTM layers for identifying temporal dependencies. Compared with the raw data as the input in LSTM model, these features are in a much lower dimensional space and thus far more concise. Therefore, inserting CNN layers ahead of LSTM layers could save significant amount of time for computation. Interestingly, even tested on a microcontroller unit of the Jetson Nano with the exact same model tested on the computer, the latency of our proposed hybrid model still maintained very short and within 1.1 ms, demonstrating a great potential to implement our developed hybrid model into predicting the pre-impact falls in real-time so that the on-demand fall protection systems (e.g., wearable airbags) can be timely activated to prevent fall-related injuries.

The present study has several limitations worth noting. First, because the SisFall dataset did not provide the video references about the simulated falls and ADLs of each subject, the pre-impact fall and fall intervals of the sensor signal labeled by authors of the baseline study may not be very consistent. Considering Xsens wearable motion capture system could record motion data and reconstruct graphical videos of human motions synchronously, we will use it to build a new fall dataset and further verify the developed deep learning algorithms. Second, for some types of falls such as a lateral fall, the duration of falling is very short and the time interval of pre-impact fall is too short to specify. Therefore, for these fall cases, pre-impact fall instances may not be labeled reliably due to the much larger width of sliding window. Further analysis on different window sizes could be conducted. Third, the development of the CovnLSTM model was based on the SisFall dataset with simulated falls performed by limited subjects. Caution is thus needed in directly applying this model into practice. Large-scale fall simulations and real-life tests with good protection need to be conducted further. Last but not least, non-fall instances are very dominant in the SisFall dataset compared with instances for other two classes, which induces challenges in training the deep learning models. More scientific techniques such as data argumentation to cope with highly imbalanced data should be explored further.

## Conclusion

We proposed a hybrid deep learning model (ConvLSTM) which integrates the CNN and LSTM architectures to predict the pre-impact fall for older people based on accelerometer and gyroscope data. The performance of this hybrid model was evaluated on SisFall, a large public dataset of various falls and ADL. We also comprehensively compared the proposed hybrid ConvLSTM model with CNN and LSTM deep learning models in terms of model accuracy, latency and learning curve. Experimental results showed that the hybrid ConvLSTM model obtained both high sensitivities (>93%) and specificities (>94%) for all three fall stages (non-fall, pre-impact fall and fall), which were higher than LSTM and CNN models. In addition, latency test on a microcontroller unit (Nvidia Jetson Nano) showed that ConvLSTM model had a short latency of 1.06 ms, which was much lower than LSTM model (3.15 ms) and comparable with CNN model (0.77 ms). High prediction accuracy (especially for pre-impact fall) and low latency on the micro board indicated that the proposed hybrid ConvLSTM model outperformed both LSTM and CNN models. These findings suggested that our proposed novel hybrid ConvLSTM model has great potential to be embedded into wearable inertial sensor-based systems to predict pre-impact fall in real-time so that protective devices could be triggered in time to prevent fall-related injuries for older people.

## Data Availability Statement

The pre-processed datasets used in this study for fivefold cross validation are available from the corresponding author upon reasonable request.

## Author Contributions

SX conceptualized the study, obtained the funding, and reviewed and edited the manuscript. HQ and SX designed the neural networks. XY did the data pre-processing and implemented the training and testing of deep neural networks. XY and HQ wrote the original draft.

## Conflict of Interest

The authors declare that the research was conducted in the absence of any commercial or financial relationships that could be construed as a potential conflict of interest.
